# Designing Lentiviral Vectors for Gene Therapy of Genetic Diseases

**DOI:** 10.3390/v13081526

**Published:** 2021-08-02

**Authors:** Valentina Poletti, Fulvio Mavilio

**Affiliations:** 1 Department of Woman and Child Health, University of Padua, 35128 Padua, Italy; 2 Harvard Medical School, Harvard University, Boston, MA 02115, USA; 3 Pediatric Research Institute City of Hope, 35128 Padua, Italy; 4 Department of Life Sciences, University of Modena and Reggio Emilia, 41125 Modena, Italy; fulvio.mavilio@unimore.it

**Keywords:** lentiviral vectors, transcriptional regulation, post-transcriptional regulation, miRNA, promoters, retroviral integration, ex vivo gene therapy

## Abstract

Lentiviral vectors are the most frequently used tool to stably transfer and express genes in the context of gene therapy for monogenic diseases. The vast majority of clinical applications involves an ex vivo modality whereby lentiviral vectors are used to transduce autologous somatic cells, obtained from patients and re-delivered to patients after transduction. Examples are hematopoietic stem cells used in gene therapy for hematological or neurometabolic diseases or T cells for immunotherapy of cancer. We review the design and use of lentiviral vectors in gene therapy of monogenic diseases, with a focus on controlling gene expression by transcriptional or post-transcriptional mechanisms in the context of vectors that have already entered a clinical development phase.

## 1. Introduction

Replication-defective retroviral vectors have been used as a convenient tool for efficient and stable gene transfer in human cells for almost three decades now. Patients suffering from severe monogenic diseases, such as immunodeficiencies, hemoglobinopathies, skin adhesion disorders and neurometabolic diseases, have been successfully treated by an ex vivo gene therapy (GT) approach based on transplantation of somatic stem cells transduced by retroviral vectors [1]. In ex vivo GT, a therapeutic gene is transferred to autologous long-term repopulating stem cells, which are then transplanted into appropriately conditioned recipients. Examples are transduced CD34^+^ hematopoietic stem/progenitor cell (HSPC) preparations or epithelial stem cell (EpSC)-derived skin grafts. According to current regulations, genetically modified cells represent “medicinal products”, produced under good manufacturing practices (GMP) and clinically developed under the same phased scheme used for conventional drugs. The first of this class of products, Strimvelis^®^, received marketing authorization in Europe in 2016 for the treatment of patients with severe combined immunodeficiency caused by adenosine deaminase deficiency (ADA-SCID) lacking an HLA-matched related stem cell donor. Strimvelis^®^ is a preparation of CD34^+^ HSPCs transduced by a first-generation γ-retroviral vector expressing an ADA cDNA under the control of the viral long terminal repeat (LTR) promoter/enhancer. Authorization was granted on data collected from 18 children treated with the product, who showed spectacular clinical benefit and a 100% survival rate on a median follow-up of >7 years [2].

First-generation γ-retroviral vectors eventually showed a high genotoxic potential, which makes them unsuited for wide clinical application. Clinical studies on three more primary immunodeficiencies, i.e., X-linked SCID, chronic granulomatous disease (CGD) and Wiskott-Aldrich syndrome (WAS), resulted in the occurrence of leukemias caused by vector-driven insertional oncogene activation [3]. These events triggered a renewed interest in retrovirus biology that led to a deeper understanding of the molecular mechanisms by which different retroviruses integrate into, and interact with, the host cell genome [4,5]. These studies were the basis for the development of a safer and more efficacious vectors, derived from the human immunodeficiency lentivirus (HIV) and carrying carefully designed transgene expression cassettes based on cellular promoters and regulatory elements [6]. A number of seminal clinical studies addressing primary immunodeficiencies [7,8,9,10], hemoglobinopathies [11,12], stem cell deficiencies [13] and neurometabolic diseases [14,15,16,17] proved the therapeutic efficacy and safety of lentiviral vectors, which eventually allowed the commercial registration of Zynteglo^®^ for gene therapy of β-thalassemia (https://www.ema.europa.eu/en/medicines/human/EPAR/zynteglo, accessed on 1 August 2021) and Libmeldi^®^ for metachromatic leukodystrophy (MLD) (https://www.ema.europa.eu/en/medicines/human/EPAR/libmeldy, accessed on 1 August 2021).

## 2. The Retroviral Genome

Retroviruses are classified in seven *genera* (α-, β-, γ-, δ- and ε-Retroviridae, Spumaviridae and Lentiviridae). They are constituted by an RNA genome, which is reverse-transcribed into a double-strand (ds) DNA molecule before being stably integrated into the host cell genome by an active mechanism mediated by the viral pre-integration complex (PIC) and the integrase protein (IN). Once integrated in the host genome, the provirus is composed of two long terminal repeats (LTRs) flanking the structural and accessory genes and sequences necessary for reverse transcription and packaging. The LTRs are tripartite elements containing essential regulatory sequences: a sequence at the 5′ end (U5) contains the polyadenylation signal, followed by a repeated sequence (R) used as primer during reverse transcription and a large sequence at the 3′ end (U3) containing the viral promoter and enhancer elements binding host cell transcription factors [18]. The U3 region plays a critical role in regulating viral gene expression and may in some cases also influence the regulation of neighboring cellular genes.

Simple retroviruses, such as γ-retroviruses, contain only four fundamental genes, *gag*, *pro*, *pol* and *env*, necessary for the viral particle production, assembly and post-entry processing eventually leading to proviral integration: *gag* encodes a precursor protein proteolytically processed into the mature MA (matrix), CA (capsid) and NC (nucleocapsid) proteins; *pol* encodes the IN and reverse transcriptase (RT) enzymes; *pro* encodes the protease (PR) that processes the gag precursor; *env* encodes the surface (SU) glycoprotein and transmembrane (TM) domains of the viral envelope, which interacts with specific cellular receptors and mediates fusion with the cell membrane. More complex retroviruses, such as lentiviruses, contain accessory genes allowing a more sophisticated regulation of viral transcription, RNA processing, nuclear entry and viral-host interactions [18].

The derivation of gene transfer vectors from retroviruses and lentiviruses essentially entails the replacement of all viral genes with a therapeutic gene expression cassette, with retention of only the sequences necessary for vector packaging, reverse transcription and integration [19,20,21].

## 3. Retroviral Integration: A Key Aspect of Viral Vector Design

Integration into the host cell genome is a non-random process crucial for the viral fitness, directed by the retroviral PIC. Different retroviruses have different integration preferences, which are maintained in the derived vectors. Integration site selection influences transcriptional regulation of the provirus and impacts on aspects like replication and latency. Integration preferences affect in turn the likelihood for a provirus to interact and interfere with transcription, splicing and polyadenylation of host cell genes, a key aspect of the safety profile of different retroviral vectors. The main viral determinants of the integration process are the PIC, with its major functional component, the IN protein, and the IN binding sites in the LTRs. The IN protein is specific to each retrovirus type and is responsible for most of its integration preferences [4,22]. Since IN is an indispensable component of a retroviral vector packaging process, it will accurately reproduce in the vector the integration characteristics of the parental virus. The PIC of most retroviruses requires a nuclear membrane breakdown to access the nucleus, resulting in efficient transduction of dividing cells only. On the contrary, a lentiviral PIC enters the nucleus by an active import mechanism mediated by specific nucleoporins and importins [23,24,25], allowing a lentivirus to transduce both dividing and non-dividing cells.

Most lentiviral vectors (LVs) are derived from HIV-1, whose integration characteristics and preferences have been extensively studied in the last decade. LVs integrate in actively transcribed genes, and their integration pattern is therefore determined by the specific transcriptional program of the target cell. High resolution maps of LV integration sites in human primary T cells and CD34^+^ HSPCs showed the LV vector preference for cell-specific, transcribed genes located in euchromatic regions in the outer, membrane-proximal portion of the cell nucleus, in close correspondence with the nuclear pore [26], supporting a functional association between nuclear entry and integration mechanisms. At the host genome level, LV PICs are tethered to their final integration sites mainly by the ubiquitous, chromatin-associated LEDGF/p75 protein [27] and integrate in transcribed gene body regions associated with specific chromatin signatures (H4K20me1, H3K36me3, H2BK5me1, and H3K27me1) [28]. This is in sharp contrast with the integration characteristics of γ-retroviral vectors, which prefer active transcriptional regulatory elements (enhancers and promoters) enriched in H3K4me1, H3K4me3 and H3K27ac markers [29]. These preferences are determined by the different nature of the chromatin factors tethering γ-retroviral PICs [30,31], and favor the disruption of host cell gene deregulation upon vector integration [5].

The ability to integrate into non-dividing cells and the safer integration profile make HIV-1 a logical choice for developing clinical gene transfer vectors. In addition, LVs offer a relatively large cargo capacity, a reduced likelihood of being silenced once integrated, and a broad choice of alternative envelope proteins for virion pseudotyping. These factors explain the almost exclusive use of LVs in the last decade to stably transfer genes into somatic stem cells and T cells for clinical applications.

## 4. Designing a Lentiviral Vector

**Figure 1:**
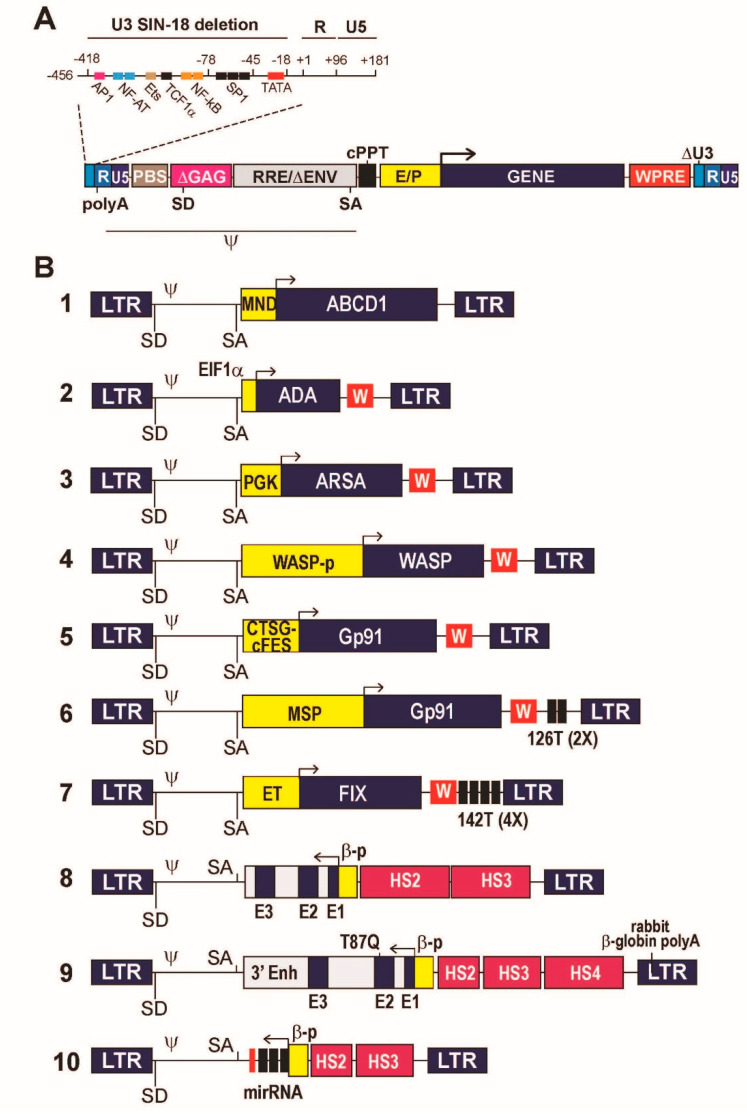
Schematic representation of HIV-1-derived lentiviral vectors (LVs) used in clinical applications of gene therapy for monogenic diseases. (**A**) A prototype, third-generation LV provirus, featuring a disabling (SIN) deletion of the enhancer/promoter sequences in the U3 region of the long terminal repeat (LTR) up to position -18 from the viral transcription start site. The enlarged portion of the LTR shows the original arrangement of the transcription factor binding sites and TATA box in the HIV-1 U3 region, deleted in the LV LTR. The poly(A) signal-containing R and the U5 regions of the LTR are retained. PBS, primer binding site; ΔGAG, deleted, non-coding portion of the GAG gene containing the D1 major HIV-1 splice donor (SD) site (CTG/GTGAGTAC); RRE, Rev-responsive element; ΔENV, deleted portion of the ENV gene containing the A7 HIV-1 splice acceptor (SA) site (TCGTTTCAG/A); cPPT, central polypurine tract; E/P, enhancer/promoter component of the expression cassette (the arrow represents the transcription start site); WPRE, woodchuck hepatitis virus post-transcriptional regulatory element; Ψ, extended packaging signal. (**B**) 1 to 10, schematic composition of the LVs described in the text. MND, modified enhancer/promoter of the murine myeloproliferative sarcoma virus; ABCD1, ATP-binding cassette, subfamily D, member 1 cDNA; EF1α, short promoter of the elongation factor 1α gene; ADA, Adenosine deaminase cDNA; W, WPRE; PGK, phosphoglycero kinase gene promoter; ARSA, arylsulfatase cDNA; WASPp, 1.6-kb extended Wiskott-Aldrich protein gene promoter; WASP, Wiskott-Aldrich protein cDNA; CTSG-cFES, hybrid promoter containing cFES regulatory regions and CTSG promoter. GP91, Gp91^phox^ cDNA; MSP, myeloid-specific promoter; 126T (2×) tandem repeat of the miR-126 binding site; ET, liver-specific transthyretin promoter/enhancer; FIX, coagulation factor IX cDNA; 142T (4×) tetrameric repeat of the miR-142 binding site; E3, E2, E1, exons 3, 2 and 1 of the human β-globin gene; βp, human β-globin gene promoter; HS2, HS3, HS4, hypersensitive site 2, 3 and 4 regions of the β-globin locus-control region; 3′ Enh, 3′ enhancer of the human β-globin gene. T87Q, codon substitution causing a tryptophan to glutamine amino acid substitution at position 87 of the β-globin protein; miRNA, gene encoding a shRNA/miR hybrid small nuclear RNA targeting the BCL11A transcription factor mRNA.

LVs are produced as replication-defective viral particles in which the RNA genome contains a so-called self-inactivating (SIN) 3′ LTR, which upon reverse transcription into a linear, double-stranded DNA genomes gives rise to an U3-deleted, enhancer- and promoter-less 5′ LTR [32]. The ΔU3 LTRs maintain a minimal, 18-bp long HIV-1 sequence necessary for integration, plus the R and U5 region containing the vector polyadenylation sequence (Figure 1A). A SIN LV provirus is thus transcriptionally inactive, allowing the vector to carry a fully independent transgene expression cassette. In addition, a LV genome maintains the cis-acting viral sequences necessary for encapsidation, reverse transcription and integration in the host cell genome, and lacks all other viral regulatory elements and genes, replaced by the transgene cargo [33]. These necessary sequences are the packaging signal (Ψ), the primer binding site (PBS) and polypurine tract (PPT) required for reverse transcription, the major HIV-1 intron with donor and acceptor splice sites, and the Rev-responsive element (RRE) required for the Rev-mediated nuclear export of the unspliced, full genomic transcript [32,33,34] (Figure 1A).

During packaging, two copies of the genomic transcript are selected from the cellular RNA pool through the interaction of the Ψ sequence, located in its 5′ untranslated region and the Gag nucleocapsid domains [35]. The lentiviral Ψ is a complex sequence encompassing part of the transactivation response element (TAR) in the U5 region, the PBS and part of the *gag* gene, all required for efficient packaging [36]. Binding of Rev to the RRE increases encapsidation up to 70-fold, making the incorporation of RRE in a LV necessary also for packaging. Overall, the size of the full LV Ψ is ∼1.7 kb [34]; attempts to remove or shorten its components resulted in low viral titers [37].

Packaging a vector genome is a crucial step in the production of infectious, replication-defective viral particles, the actual LV. This is achieved by complementation in *trans* of all necessary viral functions in the context of a packaging cell, where the genes encoding for these functions are transfected as independent plasmids. The packaging technology has evolved with time. In the first SIN LV generation system, the vector genome contained the Tat-dependent, full HIV-1 LTR and larger portions of the HIV-1 genome, and was co-transfected in the packaging cells together with two plasmids, the first expressing the *gag*, *pol*, *vif*, *vpr*, *vpu*, *nef*, *tat* and *rev* genes and a second expressing an *env* gene [38]. The HIV-1 *env* gene, encoding a glycoprotein with the HIV-1-specific tropism, was replaced by that of the bovine vesicular stomatitis virus (VSV-G), which binds the ubiquitous low-density lipoprotein (LDL) receptor [39]. VSV-G pseudotyping broadly expanded the LV tropism and usage and enabled the production of high-titer LV preparations by ultracentrifugation. Successive iterations led to the development of the so-called third-generation packaging system, devoid of all HIV accessory genes and designed to package SIN vector transcribed from a heterologous promoter—usually from the cytomegalovirus (CMV)—in a Tat-independent fashion. The system further improved the biosafety of viral production by splitting the structural *gag*/*pol* and *env* genes into two independent plasmids to reduce the chances of generating replication-competent lentiviruses (RCL) by plasmid recombination during packaging [34]. Analysis of samples from 26 different clinical trials employing third-generation LVs indicated the absence of RCL [40], confirming that this vector design significantly improves the LV biosafety and versatility for clinical applications [6,41,42].

## 5. Designing a Transgene Expression Cassette

LVs carry and express a therapeutic gene in the form of an independent expression cassette, containing in its simplest form an intron-less protein-coding region (cDNA) transcribed by a promoter and accessory regulatory regions and polyadenylated at the viral signal located in the 3′ LTR. The desired regulation of transgene expression can be obtained at the level of transcription by the use of tissue-, cell type- or differentiation stage-specific promoters/enhancers, and/or post-transcriptionally, by adding 5′ or 3′ untranslated regions (UTRs) enhancing ribosome binding or mRNA stability or target sequences for specific micro RNAs (miRNAs) to regulate protein expression by physiological RNA interference mechanisms. Viral particle pseudotyping with alternative envelope proteins has also been developed to improve transduction of specific cell types [43,44,45], but is rarely used in clinical applications where VSV-G is considered a standard for vector manufacturing.

Historically, the first LV vectors were based on the usage of strong viral promoter/enhancer sequences, typically derived from the human CMV or the murine spleen focus forming (SFFV), stem cell (MSCV) or myeloproliferative sarcoma virus (MPSV). However, LV carrying these promoters were associated with severe side effects in clinical trials, and are no longer used at least for transduction of long-lived stem cells. Side effects included insertional mutagenesis caused by the long-range, cis-acting enhancing activity of viral elements on genes flanking the provirus insertion sites, or activation of host cell defense mechanisms leading to epigenetic silencing [46]. A number of preclinical studies showed that the activity of strong viral promoter/enhancers is the most significant factor driving clonal expansion and oncogenesis upon LV integration [47,48]. Viral elements have therefore been replaced by cellular promoters and regulatory sequences in most clinical applications. The only notable exception is the LV developed for gene therapy of adrenoleukodystrophy (ALD), in which a cDNA for the ATP-binding cassette, subfamily D, member 1 (ABCD1) is driven by a modified (MND) MPSV enhancer/promoter [14,17] (Figure 1B, #1).

### 5.1. Constitutive Gene Expression

Constitutive gene expression at low-medium levels can be obtained by the use of promoters derived from human housekeeping genes. Constitutive promoters mostly used in clinical applications are derived from the translational elongation factor 1α (EF1α) or the phosphoglycerate kinase (PGK) genes. These elements showed robust safety and efficacy profile in multiple pre-clinical and seminal clinical studies of gene therapy for primary immunodeficiencies and neurometabolic diseases [49,50]. The EF1 α short promoter (EFS) has been successfully used in vectors expressing IL2RG in preclinical models [51] and clinical trials (NCT03601286 and NCT03311503) of gene therapy for X-linked SCID. In this vector design (Figure 1B, #2), protein expression was enhanced by introducing downstream from the cDNA sequence a post-transcriptional regulatory element derived from the woodchuck hepatitis virus (WPRE), which improves processing of LV transcripts [52,53]. The same design has been used in LVs for gene therapy for ADA-deficient and radiosensitive (RS) SCID. In the first case, sustained ADA expression resulted in metabolic correction, functional immune reconstitution and 100% survival in a large, multicenter clinical trial [10]. In the case of RS-SCID, however, the expression level of DCLRE1C/Artemis, a DNA repair protein, turned out to be too high both in vitro and in vivo, causing cell apoptosis and reducing the HSC repopulating capacity in an animal model of the disease. The effect was dose-dependent and was overcome by de-potentiating the vector by removing the WPRE from the expression cassette [54].

The PGK promoter has been successfully used to drive constitutive expression of the arylsulfatase (ARSA) enzyme in HSPCs for gene therapy of the neurometabolic disease MLD. Combined with a high vector copy number (VCN), the ARSA LV (Figure 1B, #3) achieved high-level expression in HSPC-derived microglia cells in the patients’ brain, and functional cross-correction of the neuronal defect in a seminal clinical trial [15,16]. An in-depth vector integration site analysis showed engraftment of a largely polyclonal population of cells and no evidence of clonal dominance in all analyzed patients [15], indicating the safety of using constitutive cellular promoters in the context of proviruses integrated in repopulating stem cells. The genetically corrected HSPCs eventually obtained marketing authorization in Europe under the commercial name Libmeldi^®^ (https://www.ema.europa.eu/en/medicines/human/EPAR/libmeldy, accessed on 1 August 2021).

An alternative to the use of an off-the-shelf heterologous promoter is the design of expression cassettes where a cDNA is driven by the natural promoter of the gene it derives from. A mandatory requirement in this case is that enough information is available on the length and composition of the sequences in addition to the minimal promoter that provides the desired level of gene expression and regulation. This normally requires a substantial pre-clinical testing in appropriate cell or animal models, possibly including the analysis of potential genotoxic effects. An example of this type of design is the LV used for gene therapy of Wiscott-Aldrich syndrome (WAS), a rare, X-linked primary immunodeficiency caused by mutations in the *WAS* gene encoding a key intracellular regulator of actin polymerization (WASP). Early attempts at gene therapy for WAS based on an MLV-derived γ-retroviral vector carrying a *WAS* cDNA under the control of the strong LTR promoter/enhancer led to insertional activation of proto-oncogenes and the development of leukemias and myelodysplastic syndromes in most treated patients [55]. On the contrary, a SIN LV expressing the *WAS* cDNA under the control of a 1.6-kb reconstituted *WAS* promoter (Figure 1B, #4) resulted in physiological WASP expression and function in the relevant blood cells, significant clinical benefit and no treatment-related adverse event in pediatric and adult patients [7,8,56,57]. Again, clonal tracking of short- and long-term repopulating HSCs by integration site analysis showed that transduction with an LV carrying a physiological promoter had no impact on cell fitness, engraftment and evolution in transplanted patients [7,58,59].

### 5.2. Cell Type-Specific Gene Expression

Restricting transgene expression to certain cell types requires the use of more complex promoter/enhancer elements or combining multiple regulatory elements into artificial promoters with the desired characteristics of transcriptional control. This type of strategy has been applied to the design of gene expression cassettes for the treatment of X-linked chronic granulomatous disease (X-CGD), a primary immunodeficiency caused by mutations in the CYBB gene encoding the gp91^phox^ catalytic subunit of the phagocyte nicotinamide adenine dinucleotide phosphate (NADPH)-oxidase, a crucial gene for the function of monocyte-macrophages. In the prototype X-CGD vector design (Figure 1B, #5), a synthetic promoter made by fusing the 5′ genomic sequences of the *FES* and *CTSG* (cathepsin G) genes allowed to restrict the expression of gp91^phox^ to the myeloid/macrophage progeny of transduced HSPCs [60]. After extensive pre-clinical validation [61], the vector is currently being used in multiple clinical trials (NCT01855685, NCT02234934, NCT02757911), which are showing safety and remarkable clinical efficacy in ameliorating the phenotype of a historically intractable disease [9]. Early attempts to treat X-CGD by expressing gp91^phox^ under the control of a strong viral promoter in the context of a γ-retroviral vector resulted in transient gene expression due to epigenetic silencing and myelodysplasia caused by insertional activation of proto-oncogenes [46,62]. No such effect was observed in the case of the X-CGD LV, again confirming the neutral role of cellular transcriptional control elements in the context of an integrated LV provirus.

A new, interesting approach to further restrict the expression of gp91^phox^ to the relevant progeny of transduced HSCs is based on the combination of a myeloid-specific promoter, generated by a fusion of 1.5 kb of CYBB upstream genomic sequence and the SP146 synthetic promoter, and a target sequence for the HSPC-specific miR126 (miRT, Figure 1B, #6). The miRNA-based regulation allowed to knock-down LV transcripts in HSPCs while permitting full expression of gp91^phox^ in monocyte-macrophages that do not express miR126. The system, that combines a transcriptional to a post-transcriptional control to restrict transgene expression to a specific cell type has been validated in a pre-clinical model [63].

### 5.3. Regulation of Gene Expression In Vivo

The miRT-based post-transcriptional control is a flexible approach to fine tune the expression of a protein encoded in an LV transgene, which can be used in multiple contexts. An important application of this technology is in preventing transgene expression in antigen-presenting cells (APCs) to minimize the immunological consequences of expressing a foreign protein in patients, thereby enhancing the applicability of LV-mediated transduction outside the context of HSC transplantation, a naturally tolerogenic environment [64,65]. One such context is the use of LVs as alternative vectors to deliver genes directly in vivo. An example is the correction of hemophilia B, a rare, X-linked blood clotting disorder caused by defective synthesis of coagulation factor IX (FIX), by expressing a FIX cDNA in liver hepatocytes by an LV delivered systemically in the peripheral blood circulation. For this application, a cDNA encoding a hyper functional FIX mutant (FIX Padua) was put under the control of the liver-specific transthyretin promoter/enhancer (ET) to restrict gene expression to hepatocytes in a vector containing four copies of the target sequence for the APC-specific miR-142 (Figure 1B, #7). Systemic delivery of FIX-expressing LV allowed stable correction of hemophilia B in murine and canine models of the disease with manageable treatment-related toxicity [66,67,68]. In dogs, the miRT-based control of FIX expression in dendritic cells and macrophages apparently prevented the appearance of anti-FIX antibodies [68]. Additionally, in a liver hepatocyte context, integration site analysis carried out in hemophilia B mice showed no evidence of clonal dominance, while the overall safety profile of the vector was demonstrated in two mouse models with enhanced sensitivity to hepatocellular carcinogenesis [68].

The use of miR-142 target sequences to control transgene expression in APCs in the context of liver-directed gene transfer was also exploited in a murine model of hemophilia A (FVIII deficiency) and a rat model of Crigler-Najjar disease (congenital hyperbilirubinemia) [69,70]. In a very different context, target sequences for miR-181a were used to restrict expression of a chimeric antigen receptor gene carried by an LV to mature T-cells by down-regulating expression in T-cell progenitors [71]. These studies showed the large spectrum of applicability of miRT-containing LVs. However, miRT-based regulation must be finely tuned to avoid off-target effects, such as miR sequestration caused by excess of miRT sequences in transduced cells with consequent loss of regulation of the natural miR targets [72,73].

### 5.4. Expression of Intron-Containing, Complex Gene Expression Cassettes

In a classical LV design, the expression cassette contains a protein-coding sequence under the form of a cDNA, often modified by adding an optimal translational initiation sequence (Kozack sequence) and optimizing codons to improve translation or eliminate cryptic splicing or polyadenylation signals. This is indeed the case for most vectors described in Figure 1B. In some cases, however, appropriate gene regulation and protein expression require sequences located in introns. In those cases, the expression cassette takes the form of a “minigene”, made by a promoter, exons, introns and a polyadenylation sequence. The minigene is then introduced in the vector in opposite transcriptional orientation with respect to that of the viral genome, which is necessary to avoid the processing of introns during transcription of the viral genomic transcript in the packaging cells.

The best examples of such a design are the vectors expressing human β-globin, developed to treat β-thalassemia and sickle cell disease (SCD) by gene therapy [74]. β-thalassemia and SCD are inherited blood disorders, respectively, characterized by defective synthesis of the hemoglobin β-chain, leading to severe anemia [75], or by synthesis of a mutated β-globin variant, the βS globin, which causes polymerization of the hemoglobin tetramers, red blood cell rigidity, vaso occlusion, anemia and multiple organ damage. In both cases, gene replacement therapy may take the form of a LV transferring an appropriately regulated β-globin gene in the patient’s HSCs and expression of normal β-globin in their erythroblastic progeny. High efficiency of gene transfer, and high levels of gene expression restricted to red cells are mandatory requirements for such an approach.

Regulation of β-globin gene expression relies on a sophisticated interplay between the β-globin promoter, upstream and downstream enhancers, and the so-called β locus control region (LCR), a large genomic region that contains enhancers and chromatin-remodeling elements that promote high-level and erythroid-restricted globin gene expression. The presence of the two β-globin introns is also essential to promote high-level globin synthesis [76]. The combination of a full LCR and a complete β-globin gene is too large to fit in an LV. Many years of pre-clinical research led to the design of two families of LVs containing reduced-size versions of the β-LCR, incorporating only the so-called DNase 1 hypersensitive sites (HS) 2, 3 and 4, and a compacted version of the non-coding portions of the β-globin gene and proximal regulatory elements (Figure 1B, #8 and 9). Both vectors showed in clinical trials transduction efficiency and β-globin expression levels sufficient to correct some forms of transfusion-dependent β-thalassemia [12,77]. Interestingly, the combination of minimal HS2, HS3 and HS4 or that of larger versions of HS2 and HS3 seemed to achieve comparable gene expression levels, while the length of the β-globin promoter and the presence of a 3′ enhancer appeared to have only a marginal impact. Due to their size and complex structure, transduction efficiency of LV globin vectors is relatively low [78], while a high VCN is mandatory to achieve phenotypic correction due to the relatively low mRNA output of a globin minigene compared to its wild-type counterpart. For this reason, correction of the most extreme forms of β-globin deficiency (homozygous β^0^-thalassemias) is only rarely achievable [12,77]. Autologous HSPCs transduced by one of these vectors (BB305, Figure 1B, #9) recently received marketing authorization in Europe for the treatment of non-β^0^ thalassemias under the commercial name of Zynteglo (https://www.ema.europa.eu/en/medicines/human/EPAR/zynteglo, accessed on 1 August 2021).

The BB305 vector contains an additional feature, a codon replacement that causes a tryptophan to glutamine substitution at position 87 in the β-globin protein sequence (T87Q) that interferes with polymerization of HbS tetramers, thus increasing its potential efficacy in the treatment of SCD. A pilot trial using this vector to treat SCD patients provided encouraging results [11], though a larger trial (NCT0214055) showed variable response and an even stronger requirement for high VCN compared to β-thalassemia [79].

### 5.5. Impact of Expression Cassette Design on Vector Titer, Performance and Biosafety

The size, and most importantly the sequence of the elements introduced in an LV have often a negative impact on vector titer and infectivity. As a general rule, the longer is an expression cassette, the higher is the likelihood that an LV will have low titer and/or infectivity. However, it is more often the complexity and the nature of the incorporated sequences rather than their length to have an impact on vector performance. During packaging, cryptic splicing and polyadenylation signals or repeated sequences inadvertently introduced in the sense strand of the LV may slow down transcription of the vector plasmid or produce defective or prematurely terminated vector genomes. Defective genomes will reduce the proportion of vector-containing virions in an LV preparation or cause defective or incomplete reverse transcription upon infection of the target cell. Reverse transcription is a crucial step in LV transduction, and any sequence that slows down or halts progression of the HIV-1 reverse transcriptase will ultimately reduce the amount of double-stranded linear genomes that can be integrated in the target cell. Defects in genome packaging or integration will read out as low titer in a conventional infectious titer assay.

To minimize this type of events, expression cassettes are normally checked for cryptic signals and repeated sequences, and eventually recoded or modified. A typical example of the impact of a defined element on vector titer and infectivity is the HS4 element of the β-globin LCR. Globin vectors containing this element consistently yield lower titers compared to vectors designed with a different combination of HS elements, independently from their overall size [80,81].

Of note, the presence of either constitutive or cryptic splicing and polyadenylation signals in an LV genome may also have an impact on its potential genotoxicity. In fact, these signals can cause aberrant splicing or premature termination of transcripts produced by a gene carrying an integrated LV, with may in turn reduce the amount of productive transcripts from the affected gene and, potentially, cause haploinsufficiency [82,83,84]. In at least one case, however, premature transcript termination caused up-regulation rather than down-regulation of a genomic transcript, and a gain-of-function effect: in a patient undergoing gene therapy for β-thalassemia, elimination of a miRNA target sequence in the 3′ UTR of the HMGA2 gene transcripts due to an LV-driven aberrant splicing event caused gene upregulation and erythroid clonal expansion [85]. The expanded clone spontaneously extinguished with time and eventually caused no adverse event [74].

### 5.6. Expression of Regulatory RNAs

A gene therapy modality alternative to gene replacement is the expression of regulatory RNAs that can modulate the expression of disease-modifying genes at the post-transcriptional level. Expression of miR-type regulatory RNAs can be obtained from LVs by designing appropriate miR gene expression cassettes driven by specific promoters. An example of such application is the up-regulation of fetal hemoglobin (HbF) expression to treat β-thalassemia or SCD. In HbF, β-globin is replaced by γ-globin, the expression of which is turned down after birth by a complex, developmentally regulated molecular switch involving specific regions of the β-globin locus [76]. A key step in this process is the repression of γ-globin expression by binding of the transcription factor BCL11A to specific target sequences [86]. Down-regulation of Bcl11A in red cell precursors reactivates γ-globin synthesis [87], which can compensate for β-globin synthesis in β-thalassemic erythroblasts or replace HbS with HbF in SCD erythroblasts, a potentially curative approach alternative to β-globin gene replacement [88]. Post-transcriptional down-regulation of BCL11A can be restricted to the erythroblastic progeny of HSCs by expressing a synthetic regulatory RNA consisting of an optimized shRNA sequence embedded within a miR scaffold (shmiR) under the control of the erythroid-specific combination of a β-globin promoter and β-LCR minimal elements [89]. A recent iteration of such LV design (Figure 1B, #10) showed safety and preliminary efficacy in a clinical trial of gene therapy for SCD, where it reactivated HbF at potentially therapeutic level [90].

## 6. Conclusions

LVs have proven their safety and efficacy as flexible gene delivery vectors in clinical applications of gene therapy for genetic diseases. Their ability to transduce both dividing and non-dividing cells and to integrate in the transduced cell genome offers the unique possibility of inserting and expressing therapeutic genes in tissues or organs maintained by dividing stem and progenitor cells, such as blood, skin, microglia or liver. Transcriptional targeting by the use of cell type-specific promoters provides the opportunity of reproducing physiological patterns of gene expression. The discovery of miRNA-based regulatory pathways has opened new potentials to the use of LVs in gene therapy. Taking advantage of the endogenous gene silencing system and the cell-type-specific expression of specific miRNAs, it is now possible to silence or modulate transgene expression in certain cell types at the post-transcriptional level, by adding short repeated miRT sequences in the expression cassette. The combination of transcriptional and post-transcriptional targeting considerably enhances the specificity of an LV-borne expression cassette, offering new possibilities such as immunomodulation of the response against foreign gene products. Genome editing is changing the landscape of gene therapy for genetic and acquired diseases. However, the biosafety profile of editing technology is still unproven, and is not necessarily going to be superior to that of LVs, which together with AAV vectors are currently the trusted workhorses in genetic medicine. Moreover, the molecular basis of a large number of genetic diseases is not necessarily or easily addressable by gene editing. Gene replacement in stem and progenitor cells is going to maintain an important place in clinical medicine in the years to come.

## Data Availability

Not applicable.
